# Inhibition of NK Reactivity Against Solid Tumors by Platelet-Derived RANKL

**DOI:** 10.3390/cancers11030277

**Published:** 2019-02-26

**Authors:** Kim L. Clar, Clemens Hinterleitner, Pascal Schneider, Helmut R. Salih, Stefanie Maurer

**Affiliations:** 1 Clinical Collaboration Unit Translational Immunology, German Cancer Consortium (DKTK) and German Cancer Research Center (DKFZ), Partner site Tuebingen 72076, Germany; Kim-Larissa.Clar@med.uni-tuebingen.de (K.L.C.); Stefanie.Maurer@med.uni-tuebingen.de (S.M.); 2 Department of Hematology and Oncology, Eberhard Karls University, Tuebingen 72076, Germany; Clemens.Hinterleitner@med.uni-tuebingen.de; 3 Department of Biochemistry, University of Lausanne, Epalinges 1066, Switzerland; Pascal.Schneider@unil.ch

**Keywords:** NK cells, platelets, cancer, immune evasion, metastasis, RANK/RANKL, denosumab

## Abstract

NK cells play an important role in tumor immunosurveillance. Their reactivity is governed by various activating and inhibitory surface receptors, which include several members of the TNF/TNF receptor family. For more than 50 years, it has been recognized that tumor immunosurveillance and in particular NK cell antitumor reactivity is largely influenced by platelets, but the underlying mechanisms remain to be fully elucidated. Here we report that upon activation, which reportedly occurs following interaction with cancer cells, platelets upregulate the TNF family member RANKL. Comparative analysis of the expression of RANK among different NK cell subsets and RANKL on platelets in cancer patients and healthy volunteers revealed a distinct malignant phenotype, and platelet-derived RANKL was found to inhibit the activity of normal NK cells against cancer cells. Notably, NK cell antitumor reactivity could be partially restored by application of denosumab, a RANKL-neutralizing antibody approved for treatment of benign and malignant osteolysis. Together, our data not only unravel a novel mechanism of tumor immune evasion mediated by platelets, but they also provide a functional explanation for the clinical observation that denosumab, beyond protecting from bone loss, may prolong disease-free survival in patients with solid tumors.

## 1. Introduction

The key role of platelets in tumor progression and metastasis has been recognized for more than 50 years [1]. Platelets interact with blood-borne tumor cells forming platelet-tumor cell aggregates, which enhance metastasis via multiple mechanisms. Beyond releasing growth factors/chemokines, facilitating endothelial adhesion and inducing epithelial-to-mesenchymal transition of tumor cells, platelets also contribute to immune evasion, another hallmark of cancer [2,3,4]. Nieswandt and colleagues observed an inhibition of metastasis formation in thrombopenic mice, while additional depletion of natural killer (NK) cells reverted this effect, suggesting that platelets guard tumor cells against elimination by NK cells [5].

NK cell effector function, which is regulated by integration of multiple inhibitory and activating signals mediated by various immunoregulatory molecules, plays a key role in controlling metastatic dissemination [6,7]. Among others, several TNF/TNF receptor (TNFR) family members influence the same [8,9,10,11]. In recent studies, we contributed to a better understanding of the mechanisms by which platelets facilitate evasion of cancer cells from NK cell immunosurveillance. This comprised analyses showing that platelet-derived TGF-β downregulates the activating receptor NKG2D on NK cells [12] and that NKG2D ligands are shed from the tumor cell surface following tumor-platelet interaction [13], thereby facilitating evasion from the NK cell “induced self” recognition mode [14]. We also reported that platelets transfer non-malignant MHC class I to tumor cells, which inhibits antitumor immunity of NK cells expressing respective inhibitory KIR receptors, thereby also impairing “missing self” recognition of tumor cells [15]. Moreover, we provided first evidence for the involvement of members of the TNF family in platelet-mediated evasion of tumor cells from NK cell reactivity: we showed that platelets transfer glucocorticoid-induced TNFR-related ligand (GITRL) to tumor cells, which results in diminished antitumor immunity due to triggering the GITR receptor that is expressed on and inhibits reactivity of NK cells [16].

Recently, other investigators reported on the expression of receptor activator of NF-κB ligand (RANKL) on TRAP6-activated platelets and its role in modulating dendritic cell function [17]. This triggered our interest since its cognate receptor RANK is expressed on NK cells and inhibits their antitumor reactivity in patients with hematologic malignancies [18,19]. Here we comparatively studied the expression of various TNF/TNFR family members on platelets and lymphocytes of cancer patients and healthy controls. We provide evidence that NK cells from patients with solid tumors display significantly enhanced RANK surface levels and that platelet-derived RANKL impairs NK effector functions against solid tumors, pointing to a role of platelet-derived RANKL in immune evasion of solid tumors from NK cell immunosurveillance.

## 2. Results

### 2.1. Expression of TNFR Family Molecules on PBMC Subpopulations

**Figure 1:**
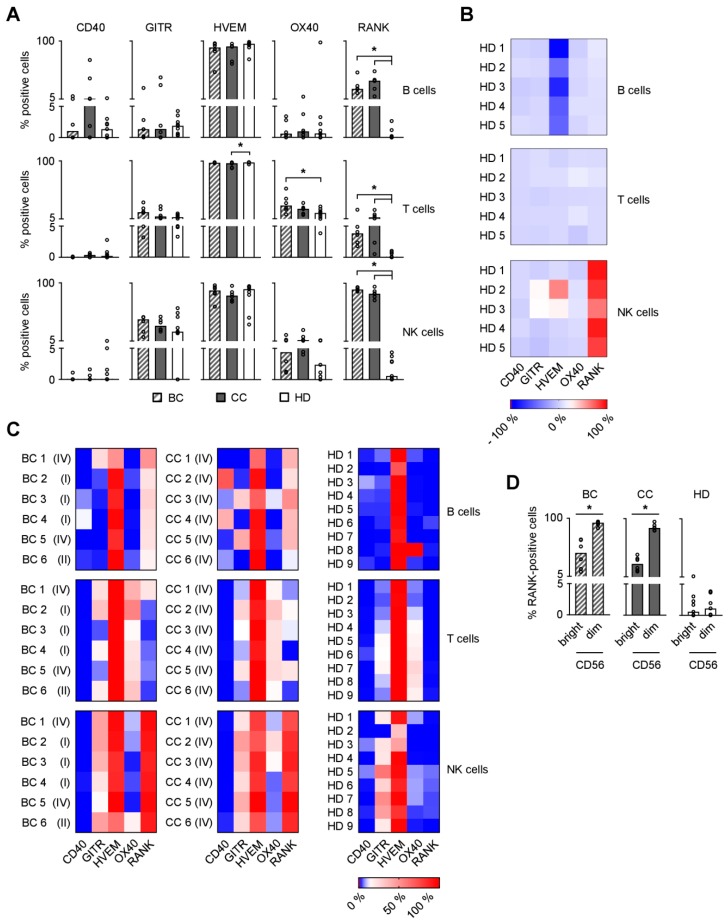
Expression of TNFR family molecules on PBMC subpopulations. (**A**,**C**,**D**) CD40, GITR, HVEM, OX40, and RANK surface expression on PBMC subpopulations from BC and CC patients and HD were investigated by flow cytometry (*n* = 6, 6, and 9, respectively). (**A**) The percentage of surface expression is indicated. (**B**) PBMC from five HD were freshly isolated or cultured without treatment for three days and CD40, GITR, HVEM, OX40, and RANK surface expression was determined by flow cytometry. Results were comparatively analyzed as follows: “percent surface expression of cultured PBMC” − “percent surface expression of freshly isolated PBMC”. The net modulation is depicted as heatmap. (**C**) Heatmap analysis of the surface expression profiles among PBMC of individual patients (disease stage as described in Table 1) and HD investigated. (**D**) The percentage of RANK surface expression on CD56^bright^ and CD56^dim^ NK cell subsets is displayed. (**A**,**D**) Median values of the respective group are depicted. Statistically significantly different results (*p* < 0.05) are indicated by *.

Members of the TNF/TNFR family are expressed on different lymphocyte subsets and influence activation, proliferation, and cell death of the respective cell types [20]. We comparatively analyzed the expression of several TNFR family molecules that reportedly influence antitumor immunity and for which antibodies with validated specificity were available, on lymphocyte populations among PBMC of patients with breast cancer (BC), colorectal carcinoma (CC) and healthy donors (HD). The clinical characteristics of the patients are given in Table 1. For CD40, no relevant expression was detected on B, T, and NK cells (Figure 1A). GITR and OX40 were expressed at intermediate levels and HVEM was expressed at high levels on all the analyzed cell populations. No profound differences were observed between patients and HD, even if biometrical analysis revealed a small statistically significant difference for HVEM in T cells of CC and OX40 in T cells of BC patients compared to HD (BC, *p* = 0.0125, ordinary one-way ANOVA with subsequent Dunnett’s multiple comparisons test; CC, *p* = 0.0206, Kruskal-Wallis test with subsequent Dunnett’s multiple comparisons test). In sharp contrast, RANK was expressed at intermediate (B and T cells) to high (NK cells) levels on all lymphocyte subsets from BC and CC patients, which differed significantly from HD who displayed only minimal RANK levels (B cells, BC *p* = 0.0069 and CC *p* = 0.0008; T cells, BC *p* = 0.0062 and CC *p* = 0.0037; NK cells, BC *p* = 0.0003 and CC *p* = 0.0160; all Kruskal-Wallis test with subsequent Dunnett’s multiple comparisons test).

To mimic the autocrine and paracrine signaling physiologically occurring among immune cells, we next determined how the TNFR expression profile of B, T, and NK cells among PBMC of HD was influenced upon culture (Figure 1B). Only minor effects on CD40, GITR, and OX40 expression were observed with all analyzed lymphocyte populations. For HVEM, a profound downmodulation was observed on B cells, while there was a slight upregulation in NK cells of some donors. Most pronounced results were again observed with RANK, for which a profound upregulation on NK cells was observed in this experimental setting.

As a next step, we investigated the specific expression patterns of the TNFR molecules on lymphocytes of patients and HD which we consider a phenotypic imprint (Figure 1C). Highly variable expression patterns among the different individuals were observed in both HD and in cancer patients. Most pronounced expression levels were observed for HVEM and RANK. While HVEM was detectable on all lymphocyte populations of patients as well as HD, RANK levels appeared to be specifically upregulated on NK cells of BC and CC patients. Of note, RANK surface levels were significantly higher on CD56^dim^ as compared to CD56^bright^ NK cells, which exert at least partially differing roles and effector functions in antitumor immunity (Figure 1D; BC, *p* = 0.0054 and CC, *p* < 0.0001; both Student’s *t* test).

### 2.2. Functional Effects of the RANK/RANKL Axis in NK Cell Reactivity Against Solid Tumors

**Figure 2:**
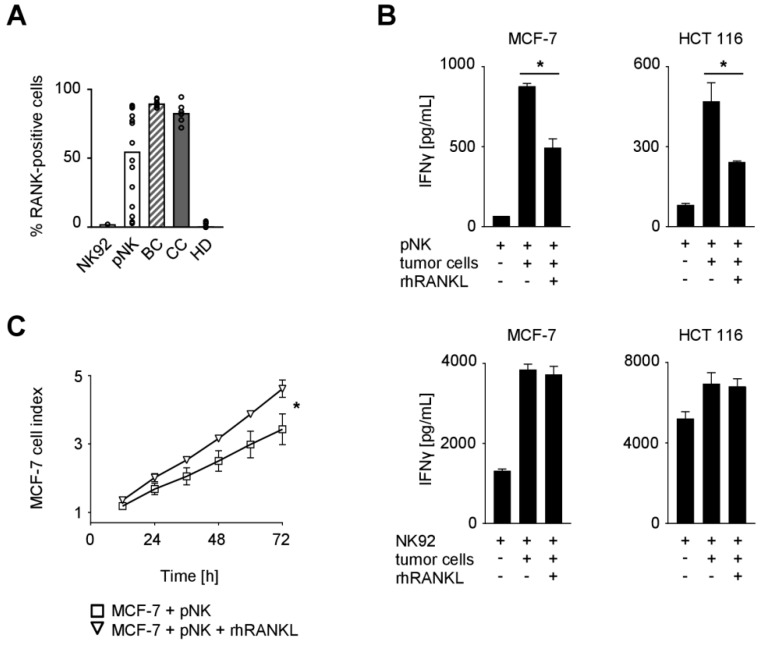
Expression of RANK and functional role of the RANK/RANKL axis in NK cell reactivity against solid tumors. (**A**) RANK surface expression on NK92 cells, pNK cells, and NK cells among PBMC from BC and CC patients and HD was investigated by flow cytometry (*n* = 1, 14, 6, 6, and 9, respectively). Median values of the respective group are depicted. (**B**) pNK (upper panel) or NK92 cells (lower panel) were cultured in the presence or absence of the indicated tumor cells and rhRANKL (125 ng/mL). IFNγ levels in culture supernatants were determined by ELISA after 24 h. (**C**) pNK cells were co-cultured with MCF-7 cells in the presence or absence of rhRANKL (125 ng/mL). The effect of NK cell reactivity on tumor cell proliferation/survival was assessed by xCELLigence RTCA for 72 h. Results are shown as electrical impedance signal (given as cell index). (**B**,**C**) Representative data of one experiment from a total of at least three with similar results are shown. Statistically significantly different results (*p* < 0.05) are indicated by *.

The analyses described above point to a potential role of RANK in NK cell-mediated immunosurveillance of solid tumors. We thus assessed RANK expression on NK cells of the cancer patients and HD as well as the NK cell line NK92 and ex vivo preactivated polyclonal NK (pNK) cells that are presently evaluated for cancer treatment [21,22,23]. While NK cells from HD, alike NK92 cells, displayed no or only minimal percentage of RANK-positive cells, BC and CC patients were found to have more than 70% RANK-positive NK cells (Figure 2A). Substantial expression of RANK was also observed with pNK cells, which were used in subsequent functional experiments since access to primary cells from cancer patients is limited. Notably, since RANK levels on pNK cells from different donors varied substantially, assays were performed with pNK cells displaying expression levels comparable to those from BC and CC patients.

To elucidate the functional relevance of RANK, NK cells were cultured with breast (MCF-7) or colorectal (HCT 116) cancer cells in the presence or absence of recombinant human RANKL (rhRANKL) to facilitate RANK triggering (Figure 2B). Analysis of culture supernatants revealed that the presence of tumor cells markedly induced IFNγ production by NK cells, which constitutes an important effector mechanism by which NK cells shape adaptive immune responses and contribute to tumor immunosurveillance. RANK signaling significantly reduced cytokine release in this experimental setting when RANK-positive pNK cells were used as effectors (MCF-7, *p* = 0.0003; HCT 116, *p* = 0.0047; both Student’s *t* test), while no effects were observed with the RANK-negative NK92 cells.

Next we determined the effect of RANK signaling on cancer cell proliferation in the presence of NK cells. To this end, MCF-7 breast cancer cells were cultured with or without pNK cells in the presence or absence of rhRANKL, which revealed significantly more pronounced tumor cell proliferation upon RANK signaling (Figure 2C; *p* < 0.0001, Wilcoxon signed-rank test). Of note, treatment of MCF-7 cells with rhRANKL alone had no relevant effects on the survival/proliferation of the breast cancer cells (Supplementary Figure S1A). These data indicate that RANK triggering protects cancer cells from NK cell attack.

### 2.3. Expression of TNF Family Members on Platelets

**Figure 3:**
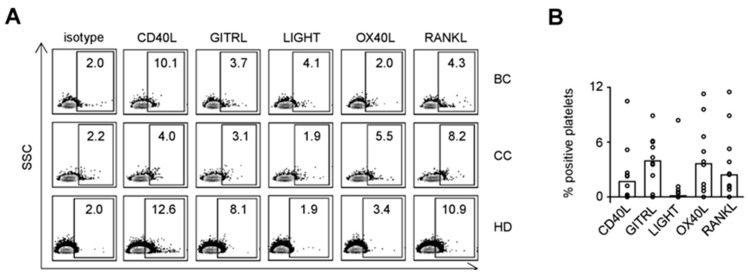

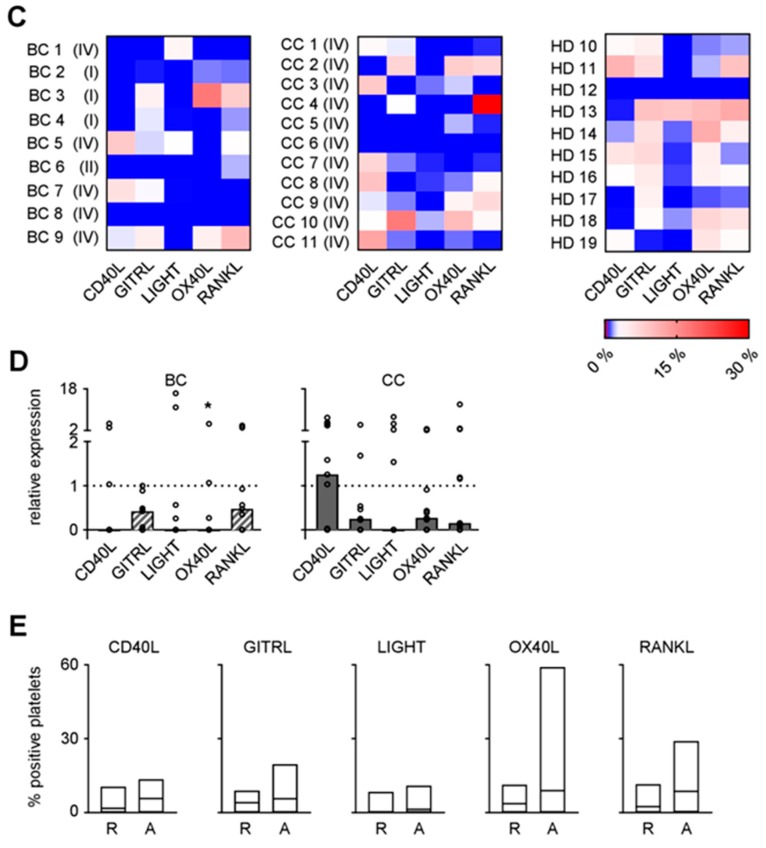
Expression of TNF family molecules on platelets. (**A–D**) CD40L, GITRL, LIGHT, OX40L, and RANKL surface expression on resting platelets from BC and CC patients and HD were investigated by flow cytometry after fixation with 2% paraformaldehyde (*n* = 9, 11, and 10, respectively). (**A**) Representative results obtained from BC and CC patients and HD are shown. (**B**) The percentage of surface expression on platelets from HD is indicated. (**C**) Heatmap analysis of the expression profiles among the platelets of individual patients (disease stage as described in Table 1) and HD investigated. (**D**) Relative surface expression on platelets from BC and CC patients compared to HD is depicted. For combined analysis, the median percentage of positive platelets obtained from HD was set to 1 for each individual TNF family molecule analyzed (dotted lines). (**E**) The percentage of CD40L, GITRL, LIGHT, OX40L, and RANKL surface expression on resting (R) or activated (exposure to thrombin for 1 min, A) platelets from HD was analyzed by flow cytometry after fixation with 2% paraformaldehyde (R, *n* = 10; A, *n* = 6). (**B**,**D**) Median values of the respective group are depicted. (**D**,**E**) Statistically significantly different results (*p* < 0.05) are indicated by *.

As platelets protect tumor cells from NK cell attack and TNFR family members like the GITR/GITRL molecule system contribute to the same, we next assessed the expression pattern of the immunomodulatory TNF family members CD40L, GITRL, LIGHT, OX40L, and RANKL on platelets from BC and CC patients as well as HD. Exemplary results are shown in Figure 3A. Combined analysis of data obtained with platelets of ten HD revealed expression levels of CD40L, GITRL, OX40L, and RANKL (Figure 3B). No relevant LIGHT expression was detected on platelets, which indicates that the LIGHT/HVEM axis may not act as major mediator of platelet-mediated immune privilege despite the high HVEM expression observed on the lymphocyte populations from BC and CC patients (see Figure 1).

Analysis of the specific expression patterns of the TNF family molecules on platelets of the individual patients and HD, considered by us as specific phenotypic imprint, revealed, alike in the analyses of the lymphocyte populations, a profound inter-individual variability (Figure 3C). When the expression of the different TNF family molecules on platelets from BC and CC patients and HD was comparatively analyzed, we surprisingly found that, except for CD40L in CC, median relative expression of all analyzed molecules was slightly lower on platelets from BC and CC patients compared to median results obtained with HD (Figure 3D; BC, OX40L, *p* = 0.0418; Kruskal-Wallis test with subsequent Dunnett’s multiple comparisons test). This is seemingly in contrast to available data that many TNF family members are upregulated on platelets following their stimulation including interaction with tumor cells [16].

To determine whether and how CD40L, GITRL, LIGHT, OX40L, and RANKL expression was influenced upon stimulation, platelets were either left untreated or were activated with thrombin (Figure 3E). Combined analysis of platelets from HD revealed no relevant induction of LIGHT and CD40L upon activation, while GITRL, RANKL and in particular OX40L appeared to be upregulated. These data point to an involvement of immunomodulatory TNF family members expressed by platelets in cancer pathophysiology.

### 2.4. Functional Effect of Platelet-Derived RANKL on NK Reactivity

**Figure 4:**
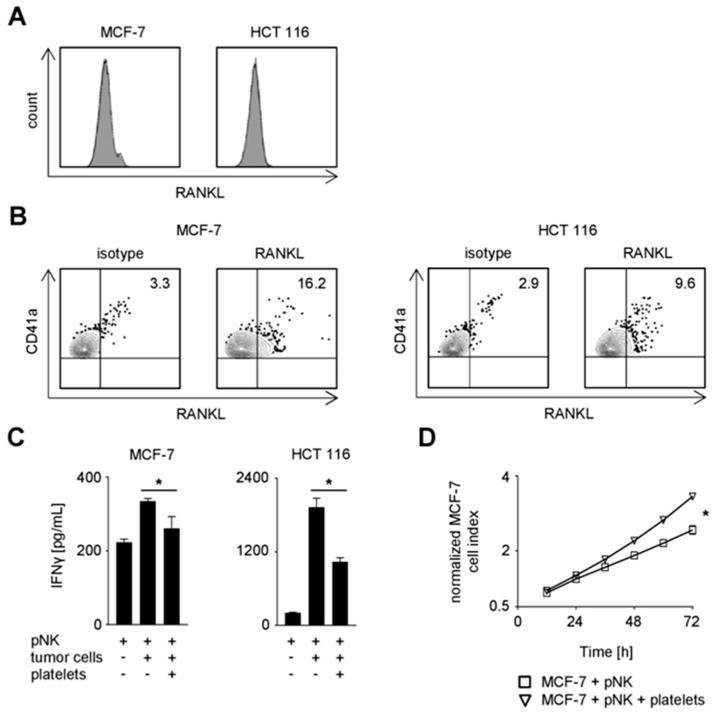

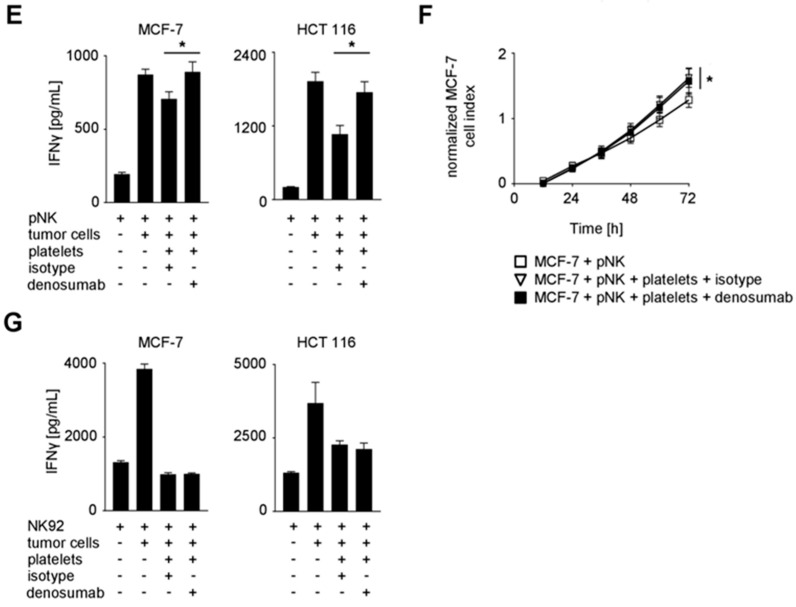
Functional role of platelet-derived RANKL in NK cell reactivity against solid tumors. (**A**) RANKL surface expression on the indicated tumor cells was investigated by flow cytometry. (**B**–**G**) The indicated tumor cells were incubated in the presence or absence of platelets from HD. Coating was performed as described in the Materials and Methods section. (**B**) RANKL and CD41a surface expression on the indicated platelet-coated tumor cells was investigated by flow cytometry. (**C**) pNK cells were cultured in the presence or absence of the indicated tumor cells and platelets from HD. IFNγ levels in the culture supernatants were determined by ELISA after 24 h. (**D**) pNK cells were co-cultured with MCF-7 cells in the presence or absence of platelets from HD. The effect of NK cell reactivity on tumor cell proliferation/survival was assessed by xCELLigence RTCA for 72 h. Results are shown as electrical impedance signal (given as normalized cell index). Cell index was normalized after addition of NK cells to the tumor cells. (**E**) pNK cells were cultured in the presence or absence of the indicated tumor cells and platelets from HD. Where denoted, denosumab (10 µg/mL) or the respective isotype control was applied. IFNγ levels in the culture supernatants were determined by ELISA after 24 h. (**F**) pNK cells were treated as indicated in (**E**) and analyzed as in (**D**). (**G**) NK92 cells were treated and analyzed as described in (**E**). (**C**–**G**) Representative data of one experiment from a total of at least three with similar results are shown. Statistically significantly different results (*p* < 0.05) are indicated by *.

Based on the expression of RANK and RANKL on NK cells and platelets of cancer patients, respectively, and the observed impairment of NK reactivity upon RANK triggering, we reasoned that RANK/RANKL interaction may contribute to the escape of cancer cells from NK cell immunosurveillance. To elucidate the specific contribution of platelet-derived RANKL in this context, MCF-7 and HCT 116 cancer cells, which lacked relevant endogenous RANKL expression, were employed in functional analyses with NK cells in the presence or absence of platelets (Figure 4A). In line with previous data by us and others [2,12,15,16], both MCF-7 and HCT 116 tumor cells were rapidly coated upon the encounter of platelets. Moreover, substantial RANKL pseudo- expression was observed on tumor cells when they were platelet-coated (Figure 4B). The presence of tumor cells induced production of IFNγ by NK cells, and this was significantly reduced when the malignant cells previously had encountered platelets (Figure 4C; MCF-7, *p* = 0.0177; HCT 116, *p* = 0.0006; both Student’s *t* test). Similarly, a protective effect of the platelets was also observed with regard to tumor cell survival/proliferation (Figure 4D; *p* < 0.0001; Wilcoxon signed-rank test). Of note, NK cell/platelet co-culture supernatants and those from rhRANKL-treated NK cells were found not to have relevant effects on the growth of MCF-7 cells (Supplementary Figure S1B).

To determine the particular relevance of RANKL in platelet-mediated tumor immune privilege, we next conducted the functional analyses in the presence of the RANKL-neutralizing antibody denosumab [24,25] (Figure 4E,F). When the effect of blocking platelet-derived RANKL on tumor cell proliferation/survival was determined, a statistically significant albeit not relevant effect of denosumab was observed as compared to the application of a respective isotype control (*p* < 0.0001; Wilcoxon signed-rank test). This was in clear contrast to the pronounced effect of RANKL-blockade on NK cell IFNγ production, where denosumab treatment restored NK cell effector function as compared to the control (MCF-7, *p* = 0.0195; HCT 116, *p* = 0.0058; both Student’s *t* test).

The effect of denosumab was in fact due to disruption of the platelet-derived RANKL/RANK axis, since no influence on NK cell reactivity was observed with the RANK-negative NK92 cells (Figure 4G). Of note, RANKL-blockade and not Fc part-mediated effects augmented NK reactivity against solid tumors as functional analyses in the presence of denosumab were found not to alter cytokine release when RANK-negative pNK cells were used in the co-culture (Supplementary Figure S2A,B). Altogether, our data demonstrate that platelet-derived RANKL impairs NK cell antitumor reactivity with a less pronounced effect on cytotoxicity compared to production of IFNγ as second major effector mechanism by which NK cells contribute to tumor immunosurveillance.

## 3. Discussion

RANKL and its cognate receptors RANK and osteoprotegerin play a key role in regulating bone metabolism [26]. Besides maintaining bone turnover in normal bone physiology, RANK also mediates osteolytic lesions in the context of metastatic cancer disease [27]. However, the RANK/RANKL axis affects cellular functions far beyond bone metabolism, among which the involvement in malignant disease, especially in solid tumors, is increasingly appreciated [19,28]. This is exemplified by the elegant work of Tan and coworkers, who showed that tumor-infiltrating regulatory T cells facilitate breast cancer metastasis via RANK/RANKL interaction [29]. Metastasis formation is a complex process which is largely influenced by various factors including the tumor microenvironment and cancer-cell-intrinsic processes, but also by platelets and immune cells including NK cells [6]. We report here that the RANK/RANKL system may influence NK cell-mediated immunosurveillance of solid tumor cells following the interaction of the latter with platelets.

In a panel of TNFR family members, RANK was found to be specifically upregulated on NK cells of patients with solid tumors as compared to healthy individuals. This is in line with previous data that various TNF and TNFR family members are upregulated on NK cells in the context of malignant disease, where they modulate antitumor reactivity upon interaction with their cognate counterparts on tumor cells [30,31]. RANKL was recently reported to be expressed on platelets, prompting us to comparatively assess the expression of this and other TNF family members on platelets of cancer patients and healthy donors [17]. The expression profile of the investigated ligands varied a lot among different patients, alike that of their respective receptors on the lymphocytes. This extends available data on the expression of TNF family molecules on platelets [32,33,34]. No difference regarding the activation level of platelets ex vivo from patients with different stages of disease was observed which may be due to the relatively small number of cases in our cohort studied. Interestingly, platelets from cancer patients displayed lower levels of the TNF family ligands despite the fact that activation, which should occur upon interaction with malignant cells, generally results in enhanced surface expression. Potential explanations for this seemingly counterintuitive finding could be provided by a reprogrammed megakaryopoiesis that occurs in cancer patients [35]. In addition, TNF family members can be shed from the ligand expressing cells following interaction with their cognate receptor [36], and this may be facilitated by the reportedly higher levels of proteolytic matrix metalloproteases detectable on platelets from patients with metastasized disease compared to healthy controls [13]. While further work is required to unravel the mechanisms, based on our observation that cancer patients display a highly patient-specific phenotypic imprint, specific expression patterns of the investigated molecules and particularly RANK/RANKL on lymphocytes and platelets, it is tempting to speculate that this “immunologic phenotype” may influence disease pathophysiology and could serve as further prognostic factor when established and correlated with disease progression upon analysis of a larger patient cohort.

Our findings that RANK triggering impaired NK cell reactivity against solid tumor cells extend our previous data on the role of the RANK/RANKL axis in hematopoietic malignancies [19]. Notably, the solid cancer cells do not have to express RANKL themselves to evade NK cell immunity [37], but rather acquire RANKL-mediated immune privilege upon coating by platelets, which facilitates activation of the latter and thus leads to increased expression of platelet-derived RANKL levels. Platelet-derived RANKL then impairs NK antitumor reactivity with a more pronounced effect on IFNγ production as compared to cytotoxicity, which is again in line with observations on the differential effect of RANK/RANKL interaction on NK reactivity in hematological malignancies. Notably, release of IFNγ by NK cells in response to tumor cells that is reinforced by denosumab treatment not only may mediate direct antitumor effects but also stimulates subsequent adaptive immune responses. In this context it is of particular interest that effects of denosumab occur very early, as confirmed by our ELISA of supernatants obtained after 24 hours of co-culture. The fact that denosumab, which is approved for treatment of osteoporosis and skeletal-related events in cancer patients, restored platelet-mediated suppression of NK reactivity not only confirmed the specific involvement of RANKL; it also provides a potential explanation for recent clinical findings obtained upon application of denosumab for prevention of skeletal-related events in breast cancer patients, which suggest that denosumab treatment may influence disease-free survival [28]. While these findings and our data certainly require additional investigation, drug repurposing of denosumab for prevention of metastatic events is appealing since it can be readily translated to the clinic due to its availability. Considering that presently multiple approaches evaluate the clinical efficacy of ex vivo expanded/activated NK cells upon adoptive transfer [21,22] the possibility to reinforce NK reactivity by RANKL blockade is particularly attractive, since we found that ex vivo expanded pNK cells express high RANK levels that, upon interaction with RANKL, inhibit their reactivity.

Altogether, we provide evidence for a new modality of platelet-mediated immune escape of solid tumors. Our findings open up new roads to use denosumab, alone or in combination with NK cell transfer, for cancer therapy, even if substantial further preclinical work is required before cancer patients ultimately may benefit from our observations.

## 4. Materials and Methods

### 4.1. Reagents

Recombinant human RANKL (rhRANKL) was from PeproTech (Rocky Hill, NJ, USA). Paraformaldehyde was from Affymetrix (Santa Clara, CA, USA). Thrombin was obtained from Sigma-Aldrich (St. Louis, MO, USA). Denosumab was from Amgen (Thousand Oaks, CA, USA). The respective isotype control was purchased from Sigma-Aldrich. Anti-CD40L antibody was from BioLegend (San Diego, CA, USA), the anti-CD40-PE conjugate was from BD Pharmingen (San Diego, CA, USA). Anti-GITR, anti-GITRL, anti-HVEM, and anti-LIGHT antibodies were from R&D Systems (Minneapolis, MN, USA). Anti-OX40 and anti-OX40L antibodies were from Ancell Corporation (Bayport, MN, USA). For staining of RANK, a recombinant Fc-RANKL was produced as described [38], the corresponding isotype control was purchased from R&D Systems and both were biotinylated with the One-step biotinylation kit according to the manufacturer’s instructions (Miltenyi Biotec, Bergisch Gladbach, Germany). Anti-RANKL antibody was from Acris Antibodies (Herford, Germany). The respective isotype controls were from BioLegend or BD Pharmingen. CD19-FITC, CD41a-PeCy5, and CD62P-FITC were from BD Pharmingen, CD3-APC/Fire and CD56-PECy7 were obtained from BioLegend. The goat anti-mouse PE conjugate was from Dako (Glostrup, Denmark), the streptavidin-PE conjugate was from BD Pharmingen. Dead cells were excluded using Fixable Aqua (Invitrogen, Carlsbad, CA, USA) after extracellular staining according to the manufacturer’s instructions. KPL TMB Microwell Peroxidase Substrate System (2-C) was obtained from SeraCare Life Science (Milford, MA, USA), Streptavidin-Poly-HRP20 Conjugate was from Fitzgerald Industries International (North Acton, MA, USA). Bicoll Separating Solution was purchased from Biochrom AG (Berlin, Germany).

### 4.2. Cell Lines

The tumor cell lines MCF-7 and HCT 116 were from German Collection of Microorganisms and Cell Cultures (Braunschweig, Germany). The NK cell line NK92MI2 (in the following named NK92) was kindly provided by K. Dennehy (Institute for Medical Virology, University Hospital Tuebingen, Tuebingen, Germany) [39,40].

### 4.3. Patients

Blood samples of patients with colorectal carcinoma or breast cancer were obtained after written informed consent in accordance with the Helsinki protocol, and the study was performed according to the guidelines of the local Ethics Committee. Patients comprised 9 cases of breast cancer and 11 cases of colorectal carcinoma. For further patient characteristics see Table 1. The study was approved by IRB (ethics committee of the Faculty of Medicine of the Eberhard Karls Universität Tuebingen) and of the University Hospital Tuebingen and was conducted in accordance with the Declaration of Helsinki; reference number 456/2017BO2, approval date was 21 September 2017.

### 4.4. Preparation of PBMC, NK Cells, and Platelets

PBMC were isolated by Ficoll/Bicoll density gradient centrifugation of blood from healthy volunteers and where indicated, cultured for three days in RPMI-1640 medium containing GlutaMAX and 25 mM HEPES supplemented with 10% FCS and 1% penicillin/streptomycin. pNK cells were generated by culturing non-plastic adherent PBMC from HD with K562-41BBL-IL15 feeder cells obtained from St. Jude’s Children’s Research Hospital (Memphis, TN, USA) as described previously [41]. pNK cells with a RANK surface expression above 70% were used for functional experiments which were performed when purity of NK cells (CD56^+^CD3^−^) was >90%. Platelets were obtained as described previously [12]. For platelet activation, 0.2 IU/mL thrombin was added to washed platelets and the suspension was shaken gently for 1 min. Subsequent fixation with 2% paraformaldehyde for 10 min was followed by two washing steps with PBS containing 1% FCS. Platelets were obtained from HD not taking any medication for at least 10 d before blood collection.

### 4.5. Treatment of Tumor Cells with Platelets

Tumor cells lines were coated with platelets as described previously with slight modifications [3,16]. In brief, tumor cells were incubated with washed platelets at a total of 50,000 platelets/µL for 30 min at 37 °C. For investigation of NK cell cytotoxicity, tumor cells were washed afterwards to remove surplus platelets and soluble factors.

### 4.6. Flow Cytometry

Flow cytometry was performed using fluorescence-conjugates or unlabeled/biotinylated mAb at saturating concentrations followed by a goat anti-mouse PE conjugate or a streptavidin-PE conjugate (both 1:100) as secondary antibodies. Analysis was performed using a FACS Canto or a FACS Fortessa (both BD Biosciences, Heidelberg, Germany). Percent positive cells were calculated as follows: “percent surface expression obtained with specific antibody” − “percent surface expression obtained with isotype control”. B cells were characterized by CD19^+^, T cells by CD3^+^, and NK cells by CD56^+^CD3^−^. Platelets were selected by CD41a^+^ and CD62P^−^ (resting) or CD62P^+^ (activated).

### 4.7. Cytotoxicity Assay

Tumor cells were co-cultured with pNK cells in the presence or absence of the indicated compounds in 96-well plates (E-Plate 96, ACEA Biosciences, San Diego, CA, USA) at 10,000 tumor cells/well. Effector to target cell ratio was 2.5:1. Real-time cell analysis was performed at 30 min intervals during a 72 h observation period using the xCELLigence RTCA system (Roche Applied Science, Penzberg, Germany). If not indicated otherwise, values are depicting means of technical triplicates with standard deviation.

### 4.8. Determination of IFNγ

IFNγ levels were analyzed by ELISA using the ELISA mAb set from Thermo Scientific (Rockford, IL, USA) according to manufacturer’s instructions. If not indicated otherwise, values are depicting means of technical triplicates with standard deviation.

### 4.9. Statistics

The 95% confidence level was used and *p*-values were calculated with a Student’s *t* test in the case of normally distributed data. Significance of not normally distributed data was calculated with a Wilcoxon signed-rank test. Multiple comparisons were performed using an ordinary one-way ANOVA for normally distributed data or a Kruskal-Wallis test for not normally distributed data with subsequent Dunnett’s multiple comparisons test.

## 5. Conclusions

Here we report on a novel mechanism of immune evasion mediated by platelets. Our data also provide a functional explanation for recent clinical observations that neutralization of RANKL by denosumab, beyond protection from bone loss, prolongs disease-free survival in patients with solid tumors as observed in breast cancer.

## 6. Patents

Helmut R. Salih and Stefanie Maurer are inventors on a patent from the University of Tuebingen that involves the role of platelet-derived RANKL in tumor immunity and its therapeutic modulation.

## Figures and Tables

**Table 1:** Patient characteristics.

BC Patients				CC Patients		
Patient Characteristics	Count	%	Patient Characteristics		Count	%
total no. of patients	9	100	total no. of patients		11	100
age (years)			age (years)			
mean	66		mean		63	
range	58–81		range		47–79	
sex of patients			sex of patients			
female	9	100	female		4	36.4
male	0	0	male		7	63.6
UICC stage			UICC stage			
I	3	33.3	I		0	0
II	1	11.1	II		0	0
III	0	0	III		0	0
IV	5	55.6	IV		11	100
receptor status			cytogenetic			
Her2	5	55.6	Ras mutation		4	36.4
HR	6	66.7	MSI		0	0
			Her2		1	9
			PIK3C		1	9
			SMAD4		1	9
			TP53		2	18
			BRAF		1	9
no. treatment regimens exposed to			no. treatment regimens exposed to			
1	5	55.6	1		2	18.2
2	1	11.1	2		5	45.4
≥3	3	33.3	≥3		4	36.4
thromboembolic event	1	11.1	thromboembolic event		2	18.2

BC, breast cancer; no., number; UICC, Union Internationale Contre le Cancer; Her2, human epidermal growth factor receptor 2; HR, hormone receptor; CC, colorectal cancer; MSI, microsatellite instability; PIK3C, phosphatidylinositol 3-kinase; SMAD4, SMAD family member 4; TP53, tumor protein 53; BRAF, v-raf murine sarcoma viral oncogene homolog B.

## Supplementary Materials

The following are available online at https://www.mdpi.com/2072-6694/11/3/277/s1, Figure S1: rhRANKL and NK cell/platelet-derived factors do not affect survival/proliferation of tumor cells, Figure S2: Denosumab does not affect NK cell reactivity via its Fc part.
