# Supplementary material for: Inhibition of NK Reactivity Against Solid Tumors by Platelet-Derived RANKL
Source: Cancers (Basel). 2019 Feb 26;11(3):277. doi: 10.3390/cancers11030277 (PMC6468810; doi:10.3390/cancers11030277)
Supplement: Supplementary file 1 [file cancers-11-00277-s001.pdf]

# Supplementary Materials: Inhibition of NK Reactivity Against Solid Tumors by Platelet-Derived RANKL

**Kim L. Clar, Clemens Hinterleitner, Pascal Schneider, Helmut R. Salih and Stefanie Maurer**

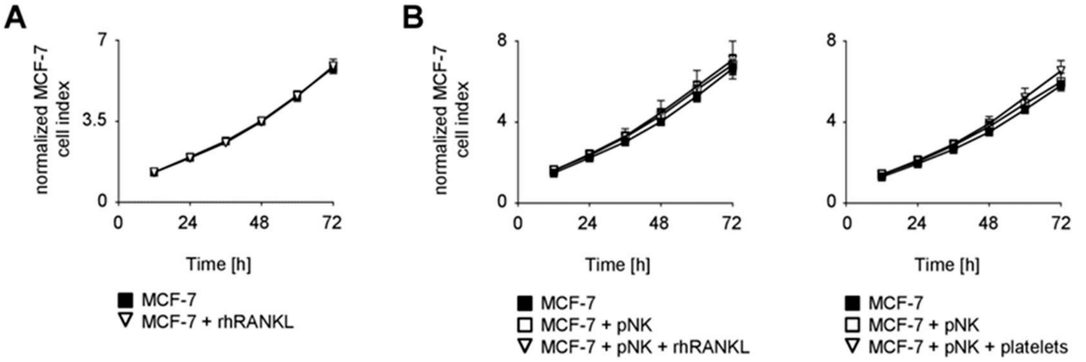


**Figure S1.** rhRANKL and NK cell/platelet-derived factors do not affect survival/proliferation of tumor cells. (**A**) MCF-7 cells were cultured in the presence or absence of rhRANKL (125 ng/mL). Tumor cell proliferation/survival was assessed by xCELLigence RTCA over 72 h. Results are shown as electrical impedance signal (given as cell index normalized after addition of rhRANKL to the tumor cells). (**B**) pNK cells were cultured in the presence or absence of 50,000 platelets/µL or rhRANKL (125 ng/mL) for 24 h. Then culture supernatants were collected and added to MCF-7 cell cultures at a ratio of 1:1. Tumor cell proliferation/survival was then analyzed as described in (A).

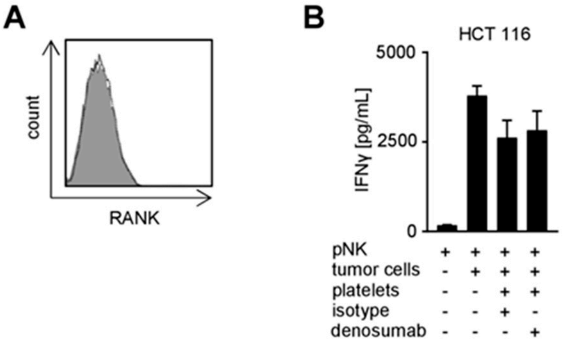


**Figure S2.** Denosumab does not affect NK cell reactivity via its Fc part. To exclude that denosumab affected NK reactivity by other mechanisms than RANKL neutralization, pNK cells lacking RANK expression as determined by flow cytometry (**A**) were cultured in the presence or absence of the HCT 116 tumor cells and platelets from HD. Where indicated, denosumab (10 µg/mL) or the respective isotype control was applied. IFNγ levels in the culture supernatants were determined by ELISA after 24 h (**B**).

© 2019 by the authors. Licensee MDPI, Basel, Switzerland. This article is an open access article distributed under the terms and conditions of the Creative Commons Attribution (CC BY) license (http://creativecommons.org/licenses/by/4.0/).
